# Smart hydrogel systems for skin fibrosis: rational design, mechanisms and therapeutic applications

**DOI:** 10.1093/rb/rbag150

**Published:** 2026-07-31

**Authors:** Ranyu Sun, Zhaojian Wang, Xiao Long

**Affiliations:** Department of Plastic and Aesthetic Surgery, Peking Union Medical College Hospital, Chinese Academy of Medical Sciences & Peking Union Medical College, Beijing 100005, China; Eight-Year MD Program, Chinese Academy of Medical Sciences & Peking Union Medical College, Beijing 100730, China; Department of Plastic and Aesthetic Surgery, Peking Union Medical College Hospital, Chinese Academy of Medical Sciences & Peking Union Medical College, Beijing 100005, China; Department of Plastic and Aesthetic Surgery, Peking Union Medical College Hospital, Chinese Academy of Medical Sciences & Peking Union Medical College, Beijing 100005, China

**Keywords:** smart hydrogel systems, skin fibrosis, biomimetic design, microenvironment remodeling, controlled cargo delivery

## Abstract

Skin fibrosis is a pathological process characterized by excessive extracellular matrix (ECM) deposition and tissue remodeling following chronic injury or inflammation. Current treatments remain limited, highlighting the need for more effective therapeutic strategies. Tissue-engineered hydrogels (TEHs) have emerged as promising platforms for fibrosis intervention due to their biocompatibility and tunable physicochemical properties. This review summarizes recent advances in TEH-based approaches for skin fibrosis, with a focus on the design of smart hydrogels. Unlike conventional scaffolds, smart hydrogels can sense pathological changes in the fibrotic microenvironment and respond to disease-associated cues, including pH changes, elevated reactive oxygen species (ROS), enzymatic activity and mechanical alterations. These adaptive systems enable controlled cargo delivery and local microenvironment regulation. We further discuss multifunctional hydrogel platforms incorporating bioactive molecules, nucleic acids and nanomaterials to modulate key fibrotic pathways. Finally, we highlight current challenges in clinical translation and future perspectives for developing safer and more effective responsive hydrogel therapies for skin fibrosis.

## Graphical abstract

**Graphical Abstract:**
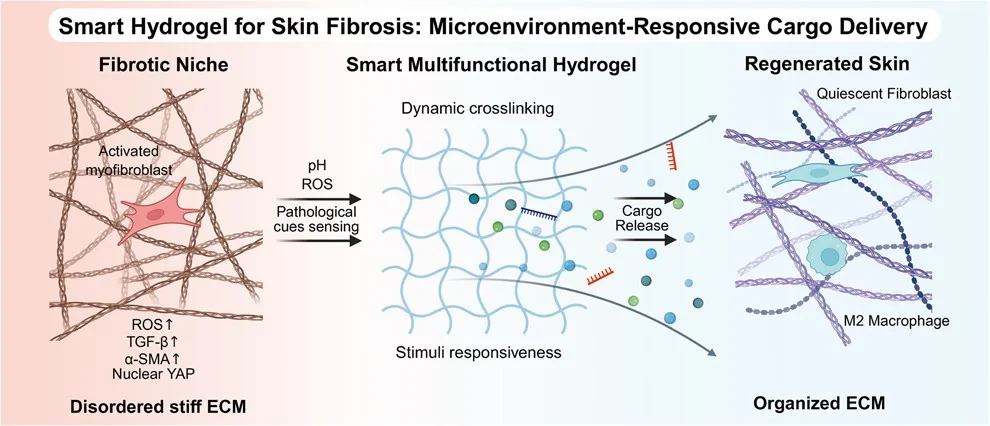

## Introduction

Skin fibrosis is a prevalent outcome triggered by various pathological stimuli, including impaired wound healing, chronic inflammation and radiation injury. Characterized by excessive deposition of extracellular matrix (ECM) and progressive disruption of tissue architecture, it manifests clinically as keloids, hypertrophic scars, systemic sclerosis and radiation fibrosis [1–3]. In addition to cosmetic defects and functional impairments, the underlying pathological network is inherently multi-mechanistic, rendering single therapeutic strategies largely ineffective and highlighting the urgent need for innovative treatment platforms. Critically, this multimechanistic network is not a static structural defect but a dynamic and self-reinforcing dialogue among fibroblasts, immune cells and the surrounding ECM, in which aberrant mechanical and biochemical cues continuously sustain fibrogenic signaling. Consequently, effective intervention demands materials that can participate in this dynamic dialogue. Therefore, biomimetic regenerative materials that mimic native ECM cues to regulate cell behavior represent a particularly well-suited therapeutic strategy for this disease.

Tissue-engineered hydrogels (TEHs) have emerged as a promising therapeutic modality for skin fibrosis, owing to their capacity to be engineered as biomimetic analogues of the native ECM rather than inert filling materials. Yet this promise is double-edged: improperly designed hydrogels can paradoxically aggravate the very pathology they aim to resolve. Key parameters, including crosslinking density, degradation kinetics, surface topography and chemical composition, directly govern hydrogel-host interactions. Excessively dense or non-degradable scaffolds, for instance, have been shown to impede cellular infiltration and provoke foreign body reactions [4, 5], while specific chemical constituents such as highly acetylated chitosan can exacerbate inflammatory infiltration and fibrous encapsulation [6], illustrating how departures from biomimetic principles cause hydrogels to be misread as foreign materials rather than constructive matrices. These observations emphasize that the therapeutic performance of hydrogels is closely linked to the extent to which their physicochemical properties recapitulate the requirements of the fibrotic niche. While biomimetic approaches have greatly advanced hydrogel design, structurally static systems remain limited in their ability to accommodate the dynamic and heterogeneous nature of fibrotic microenvironments. Smart hydrogels address this challenge by incorporating responsive elements that enable on-demand and temporally controlled therapeutic regulation.

This design imperative has driven the evolution of conventional TEHs toward smart hydrogel systems, in which biomimicry and stimuli-responsiveness play complementary roles: biomimetic motifs provide the molecular basis for recognizing the fibrotic microenvironment, while smart responsiveness translates that recognition into programmed action. In this context, a smart hydrogel system is defined as an adaptive macromolecular network that couples pathological sensing with programmed physicochemical responses, enabling it to interact dynamically with the fibrotic microenvironment rather than functioning as a passive structural scaffold. The responsive capacity of these systems spans both endogenous stimuli, including microenvironmental shifts in pH, redox state and enzymatic activity, and exogenous triggers such as near-infrared irradiation and thermal modulation. Collectively, these properties confer three defining characteristics that distinguish smart hydrogels from conventional inert matrices: the ability to dynamically adapt their mechanical properties in response to evolving tissue states, the capacity to interrupt pathological feedback loops through targeted biochemical intervention and the potential for spatiotemporally controlled multi-cargo delivery. Integrating these attributes allows smart hydrogel systems to deliver multi-targeted, microenvironment-responsive intervention against skin fibrosis, shifting the therapeutic goal from suppressing ECM over-deposition toward restoring physiologically organized tissue.

This review aims to clarify how recent material design strategies enable TEHs to construct smart hydrogel systems for the treatment of skin fibrosis. We first summarize the key design considerations for TEHs, encompassing material selection, crosslinking strategies and physicochemical property optimization. We then discuss how TEH-based smart systems regulate the fibrotic microenvironment through both biological and physical mechanisms, followed by an examination of functional modification strategies, including surface engineering, controlled payload release and cell loading, that improve therapeutic precision and efficacy. Overall, this review provides a coherent design framework and highlights the translational potential of smart hydrogel systems for anti-fibrotic therapy.

## Key design considerations for hydrogels in the treatment of skin fibrosis

### Material selection and matrix design for hydrogels

#### Biomimetic natural macromolecules as active microenvironment regulators

Hyaluronic acid (HA) and its molecular weight-dependent signaling represent a fundamental and widely utilized substrate in anti-fibrotic TEHs due to its exceptional biocompatibility and biodegradability that actively facilitates tissue remodeling [7]. Unlike inert synthetic scaffolds, native HA functions as a dynamic instructor of cellular behavior during its enzymatic and oxidative degradation *in vivo*. Its degradation products exhibit highly distinct, stage-specific functions that are strictly dependent on their molecular weight: high-molecular-weight HA (HMW-HA) actively inhibits excessive fibroblast activation and transforming growth factor-β (TGF-β) expression through CD44 receptor signaling, thereby preventing excessive collagen deposition and scar formation; conversely, the low-molecular-weight HA (LMW-HA) generated sequentially during matrix breakdown promotes healthy fibroblast migration and proliferation while guiding their ordered arrangement [8]. Due to these biological properties, HA-based hydrogels generally show good tolerance after implantation, with reduced inflammatory responses and limited fibrous encapsulation reported in soft tissue applications [9–11].

Beyond its native anti-inflammatory and homeostatic advantages, HA serves as a well-suited structural ‘chassis’ for molecular-level modulation of the fibrotic cascade. Studies demonstrate that structured HA scaffolds directly reduce fibrotic tissue thickness, inhibit macrophage adhesion and suppress type I collagen production while promoting fibroblast proliferation and healthy filopodia formation [12]. Furthermore, the abundant chemical handles on the HA backbone permit precise biochemical functionalization to target advanced signaling pathways; for instance, sulfated HA derivatives (sHA3) exhibit potent anti-fibrotic activity through a dual mechanism that competitively inhibits the formation of the TGF-β1/TβR-II/TβR-I complex, thereby directly interfering with TGF-β1–mediated dermal fibrosis progression [13]. This inherent biochemical responsiveness positions HA not merely as a passive scaffold, but as an adaptable structural chassis highly receptive to advanced engineered functionalization.

Functionalized chitosan with immunomodulatory properties represents a complementary polycationic polysaccharide matrix that exhibits structural similarities to glycosaminoglycans (GAGs) in the native ECM, making it highly advantageous for wound healing, active molecule delivery and tissue regeneration engineering. Unlike anionic polymers, chitosan functions as an active biochemical instructor during its depolymerization *in vivo*, releasing glucosamine monomers that stimulate healthy fibroblast proliferation and migration while regulating collagen deposition to prevent scar formation during soft tissue remodeling [14]. Concurrently, chitosan administration accelerates fibroblast secretion of the anti-inflammatory cytokine interleukin-10 (IL-10), which subsequently inhibits the early release of pro-inflammatory cytokines, including tumor necrosis factor-α (TNF-α) and interleukin-6 (IL-6), thereby reducing inflammatory cell infiltration [15] and decreasing pathological matrix deposition to mitigate post-wound scar formation [16]. Furthermore, the abundant hydroxyl and amino groups on the chitosan backbone provide versatile chemical handles for crosslinking, allowing chitosan-based hydrogel chassis to integrate multiple synergistic functions tailored for fibrosis mitigation [17].

In addition to these polysaccharide-based matrices, engineered collagenous scaffolds and recombinant variants provide a critical protein-based platform for preserving skin integrity and governing the biomechanical properties of the native ECM. Collagen-based hydrogels regulate cellular behavior through dynamic microenvironmental remodeling; they are progressively degraded by endogenous collagenases and matrix metalloproteinases, generating bioactive fragments and degradation peptides (derived from collagen types I, II and III) that actively recruit fibroblasts and immune cells to injury sites to initiate orderly tissue repair [18, 19]. This progressive degradation preserves the fundamental ECM architecture and regulates the dynamic equilibrium of collagen composition at skin injury sites, shifting the healing process away from functional deficits and toward normal tissue structures [19, 20]. In fact, the application of collagen-based TEHs guides wound healing such that the density, fiber size and spatial orientation of newly deposited fibers closely approach those of normal, uninjured skin [21]. Although traditional collagen implants occasionally encounter batch-to-batch variation or risk of contraction, their inherent ability to self-assemble into structured three-dimensional networks with high tensile strength, excellent cell-adhesion mediation and minimal immunogenicity makes them a premier bio-mimetic substrate for scarless skin reconstruction [22]. Recent advances in genetic engineering have enabled the development of recombinant human collagen (rhCOL), which overcomes the immunogenicity concerns associated with animal-derived collagen while promoting regenerative remodeling of fibrotic skin microenvironments through improved collagen homeostasis and reduced scar formation [7, 23].

Expanding beyond dermal structural proteins, silk fibroin (SF) scaffolds mimicking functional dermal microenvironments offer a unique natural biopolymer chassis characterized by robust mechanical performance and minimal immunogenicity across diverse regenerative medicine applications [24–26]. The structural configuration of SF effectively recapitulates the native ECM microenvironment, actively supporting cell adhesion, proliferation and rapid wound re-epithelialization [27]. Owing to their favorable and highly versatile physicochemical properties, SF matrices can be processed in the laboratory into various advanced structural form factors, including injectable hydrogels, porous membranes and electrospun nanofiber mats. These engineered morphologies significantly enhance the surface area-to-volume ratio of the scaffold and allow selective gas permeability, which facilitates continuous oxygen supply and promotes directed cell migration during targeted anti-fibrotic tissue repair [27–29].

Complementing these primary structural matrices, miscellaneous natural biopolymers with inherent anti-fibrotic potential, including cellulose [30, 31], agarose, alginate [32] and gelatin [33], have also been extensively integrated into tissue-engineering hydrogel formulations. These polysaccharides and proteins are primarily selected for their high biocompatibility, customizable mechanical performance and distinct anti-fibrotic capabilities. By acting as highly reproducible structural components or responsive blending agents, these auxiliary natural polymers modulate cell-matrix mechanical interactions and microenvironmental signaling, establishing a reliable framework to balance hydrogel bioactivity against mechanical adaptability across various stages of skin fibrosis therapy [30–33].

#### Synthetic macromolecules with tunable physicochemical properties

Natural macromolecules often provide biological cues through their native structures and degradation products. Synthetic polymers, meanwhile, offer greater reproducibility and tunable properties, and can be further modified with ECM-derived signals to enhance their biological performance.

Polyethylene glycol (PEG) with highly tunable architecture and grafting modality serves as a premier synthetic chassis to counteract the batch-to-batch variation and structural heterogeneity inherent in natural biopolymers. Due to its hydrophilic nature, well-established biocompatibility and approval by the US Food and Drug Administration (FDA) for numerous biomedical applications, and its non-degradable backbone under physiological conditions, PEG offers an exceptionally predictable structural platform for anti-fibrotic hydrogel design [34]. The primary engineering advantage of PEG-based networks lies in their highly customized macromolecular architecture, which allows for precise, mathematically reproducible control over physical properties such as crosslinking density, mesh size and macroscopic degradation rates [35]. Native PEG is biologically inert and resists non-specific protein adsorption, allowing bioactive molecules to be introduced in a controlled manner. Through diverse chemical modifications, including chain-end photopolymerization [36], block copolymerization [37] and targeted molecular grafting of cell-adhesive or anti-fibrotic peptides [38], the chemical, physical and biological profiles of PEG hydrogels can be precisely tailored to match the specific mechanics and clinical demands of fibrotic skin lesions, thereby reducing PEG’s limited biological activity and improving the scaffold’s ability to interact with disease-specific microenvironments.

To introduce biological responsiveness into similarly inert synthetic frameworks, bioactive-factor functionalized polyvinyl alcohol (PVA) for targeted therapy capitalizes on low protein adsorption and excellent biocompatibility within a highly processable synthetic polymer [39]. Unlike PEG, which often relies on end-group modification, the repeating hydroxyl units along the PVA chain allow for versatile chemical crosslinking via irradiation or glutaraldehyde, as well as physical networking through freeze-thaw cycles and crystalline self-assembly [40]. Although native PVA itself is biologically deficient and cannot inherently support cell migration or dermal adhesion, this limitation is strategically bypassed in the laboratory. By leveraging its abundant chemical handles to conjugate bioactive factors, engineered PVA hydrogels acquire precise regulatory properties, converting a passive structural barrier into an actively instructive, ECM-mimetic matrix capable of alleviating skin fibrosis [41].

Shifting the engineering focus toward biodegradable synthetic alternatives, polylactic acid (PLA) is a biodegradable thermoplastic whose hydrolytic degradation can influence the local wound environment while supporting sustained release of anti-fibrotic drugs. As the PLA matrix hydrolyzes over time, it releases its monomeric byproduct, lactic acid, which actively modulates fibroblast migration and guides collagen regeneration in close conjunction with the scaffold’s primary mechanical properties [42, 43]. To overcome the inherent hydrophobicity, brittleness and limited rheological adaptability of bulk PLA when mimicking native skin [44, 45], recent laboratory advances utilize specific microparticle formulations embedded within hydrogel networks to establish continuous, long-term drug delivery platforms [46, 47]. This encapsulation strategy substantially reduces the solubility limitations of highly lipophilic or hydrophilic anti-fibrotic compounds [46, 47]. Separately, electrospun PLA nanofiber scaffolds can be integrated to maintain stem cell bioactivity and suppress fibrotic signaling during dermal wound healing by precisely modulating the topography of the local cellular microenvironment [48].

While PLA relies on passive chemical hydrolysis, thermo-responsive Pluronic F-127 (PF-127) hybrid systems exploit an abrupt, temperature-driven physical sol-gel transition to maximize clinical injectability. Characterized by a unique nonionic amphiphilic triblock copolymer composed of poly(ethylene oxide) (PEO) and poly(propylene oxide) (PPO) blocks arranged in a PEO-PPO-PEO configuration, PF-127 exists as a free-flowing liquid at lower temperatures but spontaneously self-assembles into a three-dimensional porous network at physiological temperatures (∼37°C) due to the dehydration and micellar crosslinking of its hydrophobic PPO segments [49–51]. This sharp phase transition provides notable advantages for the low-trauma delivery and localized retention of therapeutic agents within irregular fibrotic lesions [51–59]. However, because of its low molecular weight and reversible physical interactions, pure PF-127 hydrogels suffer from mechanical instability, leading to rapid dissolution and premature drug leakage *in vivo* [60]. To preserve its thermosensitive utility while reinforcing its mechanical boundaries, PF-127 is frequently blended with biopolymers such as HA, chitosan or gelatin [61–63]. For example, combining the PF-127 chassis with sericin significantly enhances the overall mechanical strength and yields robust, stable 3D scaffolds that support prolonged cell viability and synchronized therapeutic release [59]. Although PF-127 does not provide specific biological signals, its reversible gelation enables controlled release of anti-fibrotic molecules when combined with natural polymers. Therefore, synthetic polymers such as PEG and PF-127 can provide stable material properties while preserving biological functions through appropriate modification. Ultimately, these synthetic polymers, ranging from linearly tailorable PEG to stimuli-responsive PF-127, provide highly reproducible platforms with predictable physical boundaries, establishing a robust engineering foundation for TEHs that complements the innate bioactivity of natural macromolecules.

#### Composite and hybrid systems: synergistic integration of natural and synthetic polymers

Composite hydrogels have emerged as a promising strategy to address the limitations of single-component materials by integrating the biological advantages of natural polymers with the mechanical tunability and reproducibility of synthetic polymers. For instance, although PF-127 exhibits desirable thermosensitive behavior, its relatively weak mechanical properties and limited bioactivity restrict its therapeutic applications. Incorporation of bioactive polymers such as chitosan or gelatin can improve hydrogel stability while supporting cell survival and function [61–63]. Similar synergistic effects have been achieved using oxidized carboxymethyl cellulose/chitosan systems, in which electrostatic interactions generate injectable and stable networks that facilitate scar-free wound repair [64].

Beyond simple blending, more sophisticated composite designs based on chemical conjugation and functional integration have been developed to further enhance therapeutic performance. HA-catechol conjugates combined with recombinant collagen have been shown to improve matrix elasticity and maintain collagen homeostasis [65], whereas chitosan/PEG composites enhance mechanical integrity and promote cell recruitment, resulting in prolonged stem cell retention and reduced expression of fibrotic genes [66]. More recently, nanocomposite-based approaches have expanded the functional capacity of hydrogels; for example, gelatin methacrylate matrices reinforced with dual-loaded liposomes can simultaneously strengthen the scaffold and attenuate TGF-β/Smad-mediated fibrotic signaling [67]. Collectively, composite hydrogels provide a versatile platform for balancing bioactivity, mechanical performance and manufacturing feasibility, which is essential for their future translation in fibrosis therapy.

#### Strategy for matrix selection based on specific advantages

The selection of natural or synthetic matrices for skin fibrosis therapy depends on their ability to mimic ECM, provide controllable material properties, and regulate fibrosis-related responses. Each polymer class has specific advantages rather than offering universal benefit.

Natural polymers such as HA, chitosan and collagen closely resemble native ECM in both structure and biochemical composition. Their degradation products can interact with cell receptors, such as CD44 for HA fragments, and regulate fibroblast and immune cell behavior. Meanwhile, synthetic polymers, including PEG and PVA, mainly reproduce ECM physical features, such as hydration, porosity and network structure, and usually require additional modification with bioactive molecules to improve cellular responses.

The intrinsic complexity of natural polymers can limit precise control over mechanical properties and reproducibility, whereas synthetic polymers provide more predictable regulation of stiffness, degradation and network structure through defined chemistry. However, these tunable properties alone do not directly provide the biological signals required for fibrosis modulation.

These differences determine their applications in anti-fibrotic hydrogel design. Natural polymers are often used for their inherent biological activity, such as sulfated HA-mediated inhibition of TGF-β signaling [13] and chitosan-induced IL-10 secretion [15]. Synthetic polymers are mainly used as structural or delivery platforms, enabling controlled therapeutic release through systems such as MMP-responsive PEG linkers [68] and PLA microspheres [44, 45].

From a translational perspective, natural matrices such as HA and collagen have broader clinical applications in wound healing and dermal repair, while many synthetic and hybrid systems remain under preclinical evaluation. Therefore, material selection should balance biological activity, mechanical control and clinical feasibility. Natural polymers provide intrinsic signals but often show limited reproducibility, whereas synthetic polymers offer greater tunability but require further biofunctionalization. Table 1 summarizes representative polymers, crosslinking strategies and their applications in skin fibrosis.

**Table 1:** Transitional selection strategies of hydrogel polymers based on biomimetic functions and anti-fibrotic mechanisms.

Material class	Representative polymers	Biomimetic rationale	Target anti-fibrotic mechanisms	Fibrosis-specific applications and references
Natural	Hyaluronic acid (HA)	Dermal GAG network; papillary matrix viscoelasticity.	CD44 receptor binding; TGF-β1/Smad downregulation; α-SMA suppression.	Thickness reduction [12]; fibroblast regulation [8]; TGF-β1 inhibition [13].
	Chitosan (CS)	Cationic GAG mimic; bioactive oligosaccharide signaling.	Pro-resolving M2 polarization; IL-10 secretion; migration restriction.	Inflammation attenuation [14, 15]; matrix deposition reduction [16, 17, 64].
	Collagen	Native structural backbone; integrin ligand.	Chemotaxis regulation; integration cues; contracture prevention.	Ordered dermal remodeling [18, 20, 21]; dual drug delivery [69].
	Silk fibroin (SF)	Deep dermal robust template; high O_2_ permeability.	Contraction resistance; foreign-body shielding; re-epithelialization.	Scar-inhibiting bilayer scaffolds [27, 68]; sustained cargo release [26, 28, 29].
Synthetic	Polyethylene glycol (PEG)	Chemically defined chassis; highly tunable mesh compliance.	YAP/TAZ mechanotransduction disruption; stable low-stiffness boundary (3–10 kPa).	Protease-cleavable responsive networks [70]; stem cell niche delivery [71, 72].
	Polyvinyl alcohol (PVA)	Hydrogel hydration mimic; fetal-like scarless microenvironment.	Protein fouling prevention; early inflammatory spike suppression; tension shielding.	Compliance tuning in hybrid systems [73, 74]; surface bio-functionalization [41].
	Polylactic acid (PLA)	Long-term deep support; microenvironmental pH buffering.	Proliferation restriction via slow acidic degradation; contracture counteraction.	Anti-fibrotic small-molecule delivery [71, 75]; stem cell stabilization [46–48].
	Pluronic F-127 (PF-127)	Shear-responsive transition; transitional granulation matrix.	Temperature-induced gelation; zero-shear irregular gap sealing.	Pathway inhibitor thermogels [76]; cell-free exosome translation [59, 77–80].

### Crosslinking architectures in hydrogel design

By selecting specific crosslinking strategies, the properties of composite hydrogels, formed from multiple base materials, can be precisely controlled. These properties dictate the hydrogel’s interaction with the implantation site and guide stem cell differentiation via adhesion kinetics and surface morphology.

#### Covalent crosslinking networks

Covalently crosslinked hydrogels are widely used for stem cell encapsulation. By forming covalent bonds between polymer chains, they create tunable systems that allow precise control over mechanical properties, degradation kinetics, biocompatibility and structure. Common strategies include radical polymerization, chemical crosslinking and hybrid methods [81].

Crosslink density critically regulates cellular behavior. In covalently crosslinked HA hydrogels, human mesenchymal stem cell (hMSC) fate is guided by traction forces generated during hydrogel degradation, and suppressing these forces by delaying secondary crosslinking inhibits differentiation [82]. Mild HA-polyisocyanurate (PIC) crosslinking promotes hMSC proliferation and spreading while conferring anti-fibrotic properties [83]. Although optimized covalent crosslinking enhances mechanical properties and adhesion strength, better mimicking tissues like skin [84], excessive crosslinking impedes cell spreading. Moreover, direct mixing to induce strong interactions fails to produce TEHs with desired biocompatibility, conformability and injectability [85].

#### Non-covalent crosslinking networks

Physical crosslinking primarily relies on reversible non-covalent interactions, such as electrostatic forces, hydrogen bonds, hydrophobic interactions and host-guest recognition. These weak interactions impart self-healing, stimulus-responsiveness [86, 87] and enhanced mechanical integrity to hydrogels [88]. They also confer additional functionalities, including strong adhesion, antioxidant and antibacterial activities [89]. These dynamic systems adapt to pathological skin microenvironments, enabling stage-specific treatment that mitigates fibrosis, reduces scarring and promotes regeneration.

Weak interactions can be leveraged to engineer hydrogels with tailored therapeutic properties. In one strategy, the synergistic physical interaction between HA-catechol and rhCOL enhances hydrogel elasticity and adhesion by dissipating mechanical energy. This helps maintain balanced collagen deposition during wound healing, preventing fibrosis [65]. Alternatively, introducing hydroxyl and carboxyl groups can counteract the strong electrostatic forces in systems like oxidized carboxymethyl cellulose sodium (O-CMC) and chitosan (CS). This approach promotes the formation of uniform, injectable and stable hydrogels that help minimize scarring [64].

#### Emerging dynamic covalent crosslinking strategies

To bridge the gap between permanent covalent networks and weak physical interactions, dynamic covalent chemistry (DCC) has been widely exploited in anti-fibrotic hydrogel design. Unlike physical interactions, dynamic covalent bonds, most notably dynamic Schiff bases [64], involve true chemical orbital overlap yet exhibit reversible cleavage and re-formation under physiological conditions. For instance, amino-aldehyde dynamic Schiff base hydrogels display inherent shear-thinning and self-healing behaviors, adapting seamlessly to irregular skin wound beds [64, 66].

Recent studies demonstrate that these dynamic networks combat skin fibrosis through mechanical modulation, drug delivery or cell transplantation. In one approach, a thermo-responsive Schiff base hydrogel exhibits 20% active contraction, matching skin elasticity and substituting for myofibroblast contraction to break the tension-driven fibrotic loop [75]. Concurrently, its progressive degradation enables controlled drug release, downregulating TGF-β1-induced α-SMA expression and reducing scar area by 40% with balanced type I/III collagen [75]. Alternatively, these dynamic networks serve as protective vehicles for cell therapy. An injectable chitosan/PEG hydrogel extended mesenchymal stem cell retention to 10 days, upregulating anti-inflammatory factors while downregulating pro-fibrotic Col1A, Col3A, α-SMA and TGF-β expression and increasing the Mmp1/Timp1 ratio for matrix remodeling [66]. Thus, dynamic covalent crosslinking provides a versatile anti-fibrotic platform.

Taken together, the choice of crosslinking strategy, much like material selection, depends on the balance between mechanical stability and adaptability. Covalent networks provide durable mechanical support and predictable degradation, making them suitable for long-term applications, but their fixed structure limits responsiveness to changes in the fibrotic microenvironment. Physical and dynamic covalent crosslinking offer greater reversibility and self-healing capacity, allowing hydrogels to better accommodate ECM remodeling and wound progression. Therefore, the optimal crosslinking strategy should be selected according to the specific requirements of the intended application.

## Enhancing hydrogel physicochemical properties for advanced application

**Figure 1:**
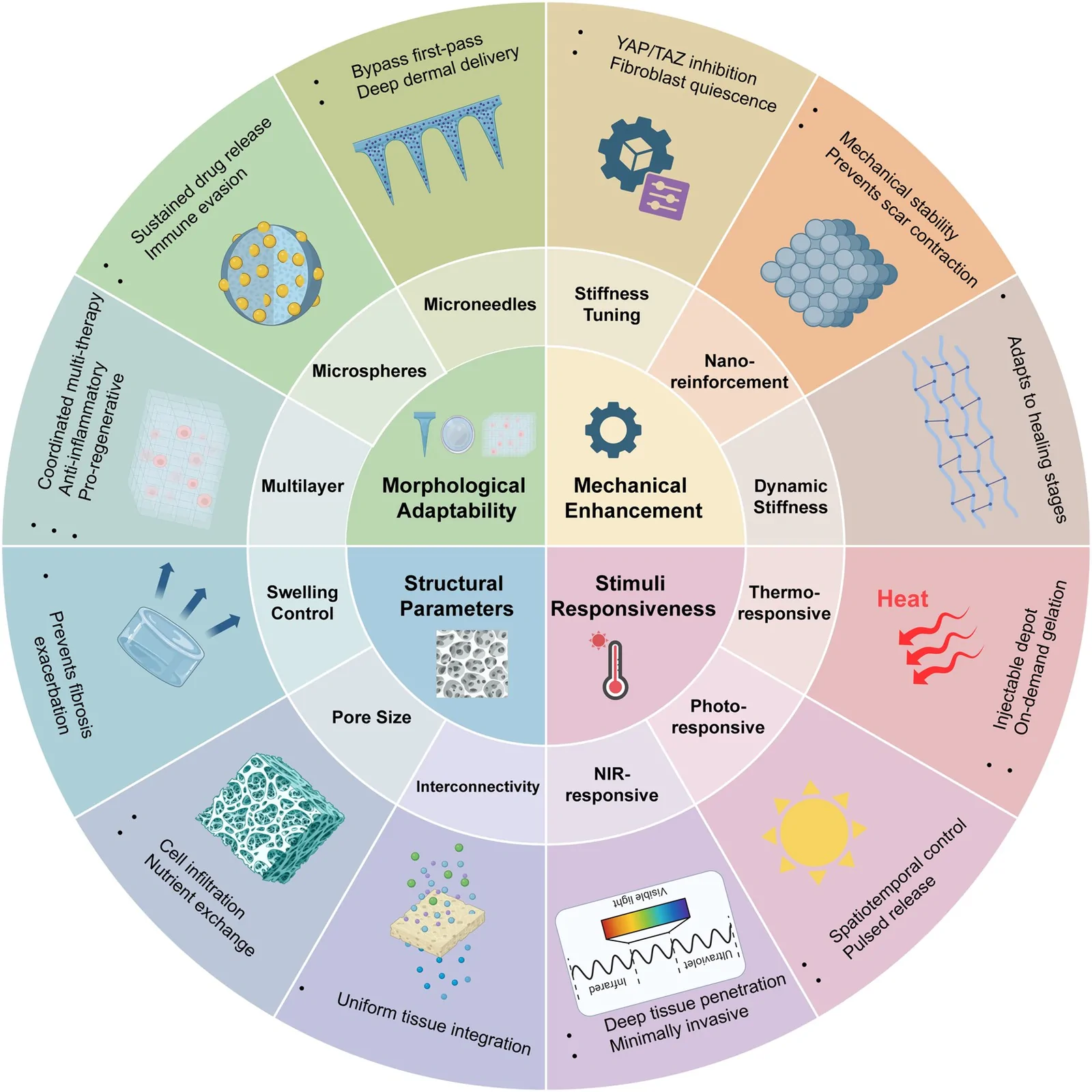
Hydrogel physicochemical property enhancement strategies enable precise modulation of the fibrotic microenvironment through tunable mechanics, stimuli-responsiveness, structural control and morphological adaptability.

Hydrogel physicochemical properties, such as mechanics, stimulus responsiveness and microstructure, critically influence skin integration, cell behavior and therapeutic delivery. Consequently, optimizing these properties is essential to advance hydrogels from passive scaffolds to biomimetically instructive, active anti‑fibrotic platforms. The following section examines key design strategies, including mechanical stiffness, thermal/photo‑sensitivity and pore morphology, highlighting how each contributes to the prevention and reversal of skin fibrosis. A graphical summary of these enhancement strategies and their therapeutic implications is presented in Figure 1.

### Mechanical strength and stiffness

Mechanical stress and stiffness are among the key characteristics determining the mechanical properties of hydrogels. They serve as critical factors in achieving mechanical compatibility with skin tissue and influencing cellular fate. The elastic modulus of the dermis is typically observed to range between 3 and 10 kPa. This range is considered conducive to maintaining the normal phenotype of fibroblasts and promoting regeneration.

Nano-reinforcement strategies enhance hydrogel mechanics mainly through two approaches: physical filler incorporation and crosslink density modulation. The incorporation of nano-reinforcing agents like cellulose nanocrystals (CNCs) significantly improves the mechanical stability of hydrogels while maintaining their softness [90]. In cyclodextrin/polymer inclusion complexes, cellulose nanowhiskers (CNWs) enhance gelation, strengthen the network and enable controlled drug release [91]. Similarly, CNCs combined with carboxymethyl chitosan (CMC) increase crosslink density and structural integrity [92]. Dialdehyde cellulose nanocrystals (DACNCs) further help preserve the system’s crystalline structure, improving gel strength and enabling self-healing properties that promote deep wound healing with reduced scarring [93]. Recent studies have also shown that nanocomposite hydrogels with enhanced mechanical properties can regulate the wound microenvironment and reduce scar formation. For example, a gelatin methacrylate-based hydrogel reinforced with dual-loaded liposomes improved mechanical stability while reducing inflammation, oxidative stress and TGF-β/Smad-mediated fibrosis, highlighting the role of mechanical optimization in scarless wound repair [67]. Beyond static reinforcement, dynamically tunable stiffness systems using ion crosslinking or Schiff bases can mimic the mechanical gradients of healing tissue, allowing real-time regulation of cellular mechanical signals and paving the way for a programmed mechanical environment. Collectively, these strategies enable hydrogel stiffness to be tuned within the physiologically relevant 3–10 kPa range noted above, helping to maintain a fibroblast-compatible mechanical niche while resisting the pathological matrix stiffening that drives myofibroblast activation.

### Thermo-responsivity and photo-responsivity

Thermo-responsive and photo-responsive hydrogels can alter their physical state or mechanical properties in response to external stimuli, improving their adaptability in fibrotic tissues. A key example is the thermo-responsive polymer PF-127, which undergoes a temperature-dependent sol-gel transition, remaining liquid at 4°C and forming a gel near 37°C. This thermo-responsive behavior enables localized formation of hydrogel networks for the treatment of superficial skin fibrosis [59, 77, 94].

Thermo- and photo-responsive systems mainly serve as physical approaches to regulate hydrogel formation and structural properties. Thermo-responsive PIC gels, for example, provide a reversible gelation process that maintains a suitable cellular environment and can be removed by cooling, reducing additional tissue damage and fibrosis [95, 96]. This reversible physical transition offers an advantage over permanently crosslinked systems when repeated adjustment or removal is required. Photo-responsive systems such as photo-crosslinked gelatin methacryloyl (GelMA) allow light-controlled regulation of gelation kinetics and network formation by adjusting light intensity and exposure time [97]. These thermo- and photo-responsive strategies are therefore primarily used to control hydrogel formation, mechanical properties and structural adaptation in fibrotic tissues.

Table 2 summarizes the key stimulus types along with their responsive moieties, typical hydrogel systems and physical-state outcomes relevant to anti-fibrotic applications.

**Table 2:** Physical stimulus-responsive systems for hydrogel state modulation in skin fibrosis applications.

Stimulus type	Responsive moiety/mechanism	Typical hydrogel	Physical-state outcome	Trigger condition	Clinical advantage
Thermal	Temperature-dependent sol-gel transition	Pluronic F-127; polyisocyanide (PIC) gels	*In situ* gelation; reversible removal by cooling	Body temperature (∼37°C)	Minimally invasive delivery [59, 77, 78]; tissue-damage-free removal [95, 96]
Photo (crosslinking)	Photo-crosslinking; light-regulated network density	Photo-crosslinked GelMA	Tunable gelation rate and stiffness	External light irradiation (intensity and duration controlled)	Spatiotemporal control over scaffold formation [97]

### Morphological engineering of hydrogels: from microscopic pores to macroscopic patches

The therapeutic efficacy of hydrogels in managing skin fibrosis is dictated by their structural morphology across multiple length scales. Rational structural engineering, from microscopic pores to macroscopically tailored shapes, enables hydrogels to interface with diverse wound beds, modulate mechanotransduction and sustain localized therapeutic delivery.

#### Microscopic structural geometries

At the microscopic level, hydrogel pore size, connectivity and porosity control cell infiltration and drug release [46, 98–100]. For instance, a 6%:5% gelatin-PVA formulation with large, interconnected pores (>50 μm) can increase the infiltration of fibroblasts, thereby accelerating structured wound healing and reducing fibrosis [73]. Techniques such as 3D printing allow precise engineering of these parameters to create biomimetic microenvironments that guide orderly cellular alignment.

Furthermore, rational pore design balances hydrogel swelling for exudate management and drug release, as excessive swelling can lead to tissue compression and secondary fibrotic progression [101]. An optimal swelling-release balance can be achieved by modulating crosslinking density and matrix hydrophobicity. This is exemplified by the 4-formylbenzoic acid-grafted PF127 (PF127-CHO)/carbonic dihydrazide-modified gelatin (Gel-CDH) (P&G) hydrogel, whose densely crosslinked dynamic network suppresses excessive swelling, prevents adverse tissue compression, and sustains localized anti-fibrotic drug release [75].

#### Macroscopic morphologies

To expand clinical utility, macroscopic hydrogels are engineered into distinct form factors tailored to specific pathological skin conditions.

As a particulate morphology, injectable microspheres provide uniform mechanical properties and can readily conform to irregular and deep fibrotic wound beds, allowing minimally invasive filling with reduced tissue damage. For example, an ACEI-loaded PLGA microsphere-hydrogel system limits swelling to maintain structural integrity while providing sustained anti-inflammatory drug release and reducing collagen deposition [75]. Similarly, a multilayer injectable system (SA/BG-SACM-PLGAPFD) incorporates hydrophilic and hydrophobic microspheres into the ECM to achieve sustained co-delivery of multiple anti-fibrotic agents [102]. These features make injectable microspheres particularly suitable for deep or irregular lesions, such as radiation-induced skin fibrosis, where topical formulations cannot provide adequate tissue coverage.

Microneedle (MN) systems overcome the barrier of the fibrotic stratum corneum and deliver therapeutics directly into the dermis, improving local drug delivery [103–106]. For example, core–shell MNs loaded with siSPARC nanocomposites suppressed secreted protein acidic and rich in cysteine (SPARC) expression and reduced collagen deposition [107]. Luan *et al.* [108] developed an MN platform that co-delivered paeoniflorin to reduce the toxicity of triptolide while enhancing its anti-fibrotic effects and improving collagen organization. In addition, near-infrared (NIR)-responsive MNs enable controlled release of anti-fibrotic agents and inhibit TGF-β1/α-SMA signaling to alleviate skin fibrosis [109]. These systems are particularly useful for treating hypertrophic scars and keloids, where the thickened stratum corneum limits passive drug penetration.

For larger treatment areas, multilayer hydrogels and topical patches are designed to mimic the layered structure of native skin and provide localized anti-fibrotic therapy [68, 110, 111]. A bilayer SFMA/GelMA hydrogel includes a HaCaT-loaded upper layer to promote epithelialization and a GelMA/HA methacrylate (HAMA) lower layer that delivers antimicrobial and pro-angiogenic factors to reduce fibrotic scarring [112]. Similarly, a dual-layer AM/ESF/AT-MSCs hydrogel consists of a decellularized amniotic membrane (AM) outer layer that acts as an anti-inflammatory barrier and an electrospun sericin (ESF) inner layer that regulates TNF-α/TGF-β1 signaling and reduces abnormal collagen deposition [113]. Such multilayer patches are well-suited for covering extensive superficial lesions, including post-burn hypertrophic scars and large cutaneous plaques in systemic sclerosis.

In summary, controlling hydrogel morphology across multiple scales improves tissue integration, regulates cell-matrix interactions and enables localized therapeutic delivery. These structural features allow hydrogels to better adapt to different wound environments and support effective anti-fibrotic repair.

Mechanical properties, responsiveness and morphology each contribute to hydrogel performance in different ways. Mechanical optimization influences how cells sense and respond to their surroundings; responsive systems improve control over therapeutic delivery and structural design facilitates tissue integration and localized treatment. Together, these properties allow hydrogels to participate actively in tissue repair rather than simply serving as passive scaffolds. Building on these physicochemical features, the next section focuses on how hydrogels regulate cellular and molecular processes involved in fibrosis.

## The mechanisms of hydrogel in the treatment of fibrosis

**Figure 2:**
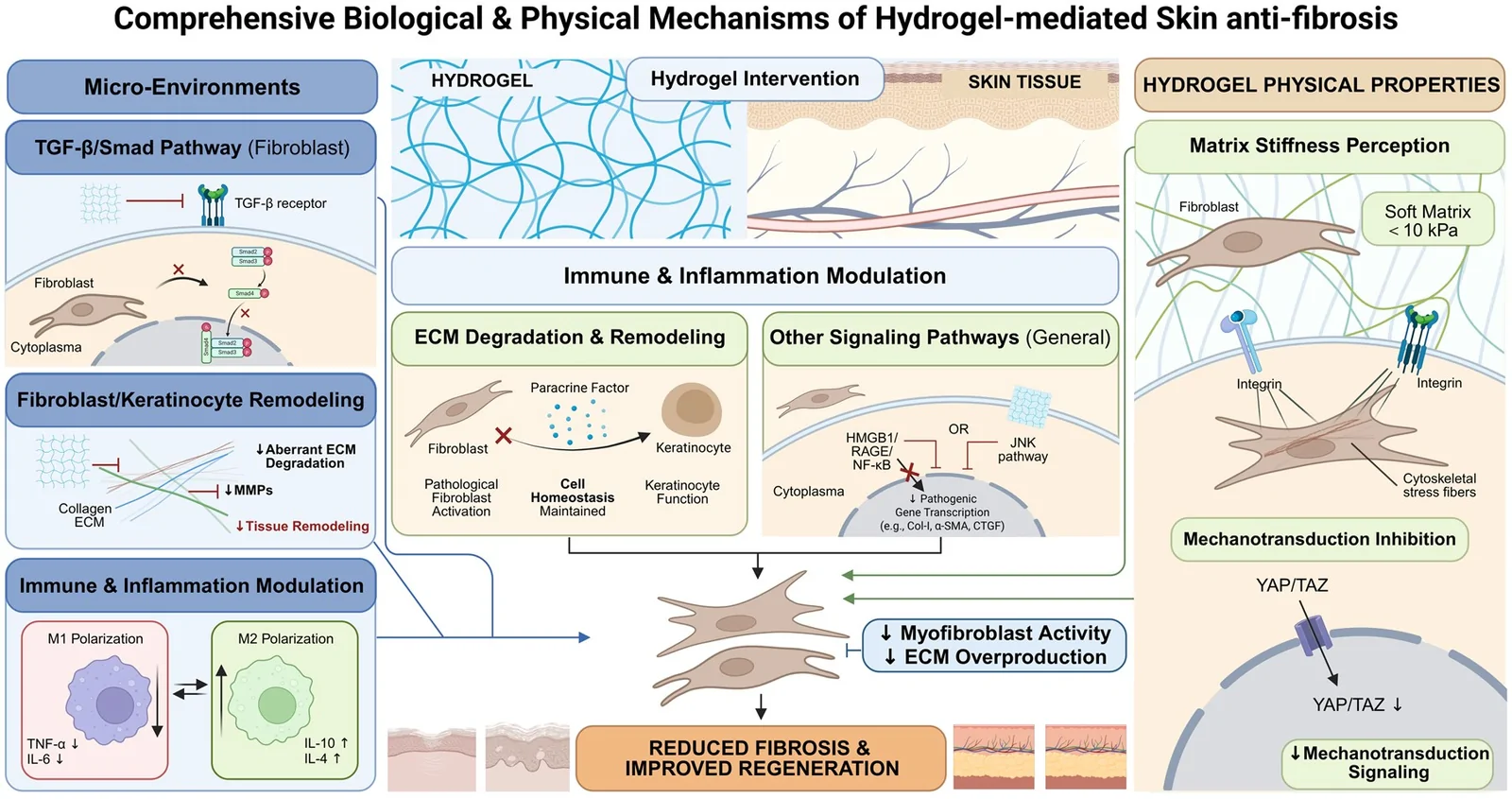
Comprehensive biological and physical mechanisms of hydrogel-mediated skin anti-fibrosis. (Left) Biological pathways regulate TGF-β/Smad signaling, cellular homeostasis, and macrophage M1-to-M2 polarization. (Right) Physical pathways target mechanotransduction, with a soft matrix (<10 kPa) suppressing integrin activation and YAP/TAZ nuclear translocation. (Bottom) Both pathways converge to inhibit fibroblast pathogenic gene transcription, decreasing myofibroblast activity and ECM overproduction to promote skin regeneration.

Skin fibrosis is a complex process driven by multiple signaling pathways, leading to aberrant fibroblast activation, excessive ECM deposition and a disrupted mechanical microenvironment. Beyond their role as physical scaffolds, TEHs enable multi-level interventions by modulating biochemical and mechanical signals within this pathological niche. By incorporating biomimetic features, these strategies have shown potential for reducing fibrosis and limiting scar formation. The underlying mechanisms, encompassing both biochemical and physical pathways, are summarized in Figure 2 and detailed in the subsequent sections.

### Biological mechanisms

#### Modulating the TGF-β/Smad pathway

The TGF-β/Smad axis is a principal pathway in skin fibrosis, driving fibroblast-to-myofibroblast differentiation via SMAD2/3 phosphorylation [114]. This universal mechanism, confirmed in multiple hydrogel studies [67, 115, 116], further promotes epithelial-mesenchymal transition (EMT)/EndMT through TWIST and SNAIL, transforming epithelial and endothelial cells into a fibroblast-like state. These events, coupled with suppressed matrix metalloproteinase (MMP) activity and fibrinolysis due to elevated tissue inhibitor of metalloproteinases (TIMP) and Plasminogen activator inhibitor-1 (PAI-1), create a self-reinforcing cycle of excessive ECM deposition [117].

To counteract this, TEHs can be loaded with targeted inhibitors or bioactive factors that directly suppress, indirectly regulate or provide feedback modulation of the TGF-β pathway, offering a promising strategy to treat skin fibrosis.

1. **Direct inhibition** 
  This strategy employs hydrogels that function by directly binding and neutralizing TGF-β, thus inhibiting its pathway. A key example is highly sulfated HA (sHA3), which inhibits TGF-β1 signaling by blocking its receptor complex, thereby reducing Smad2 phosphorylation and mitigating skin fibrosis [13]. Other hydrogels incorporate specific inhibitors, such as the retinoid TP [108] or TGF-β-binding peptides [118], to achieve the same therapeutic goal.
2. **Indirect inhibition** 
  Hydrogels can also suppress TGF-β/Smad signaling indirectly by influencing upstream or downstream regulators. For example, vitamin A-conjugated liposomes selectively deliver drugs to (retinol-binding protein 1) RBP1-expressing myofibroblasts, while co-delivered heat shock protein 47 (HSP47) small interfering RNA (siRNA) disrupts collagen folding and induces endoplasmic reticulum stress, promoting myofibroblast apoptosis [119]. Similarly, a triple-layered PCL-chitosan/PVA-metformin scaffold continuously releases metformin to activate the AMP-activated protein kinase (AMPK) pathway, thereby inhibiting TGF-β1/Smad signaling and producing combined anti-fibrotic and regenerative effects [74].
3. **Feedback regulation** 
  Some polysaccharide-based hydrogels suppress TGF-β signaling by modulating reactive oxygen species (ROS) and restoring redox balance, thereby interrupting its self-amplifying feedback loop. The COS@Mn-MSN nanocomposite integrates Mn-mediated ROS scavenging, Si-driven macrophage polarization, and the anti-inflammatory activity of COS to reprogram the wound microenvironment. This coordinated regulation attenuates the ROS/TGF-β1/Smad7 axis and inhibits fibrosis [120].

Beyond single-mechanism approaches, TEHs can also integrate dual strategies for enhanced efficacy. Coentro *et al.* [69] developed a core-shell collagen hydrogel wherein the core contains a TGF-β trap (T122bt) to directly neutralize TGF-β, and the shell incorporates the histone deacetylase (HDAC) inhibitor TSA to prevent myofibroblast transformation. This coordinated system simultaneously targets TGF-β signaling and collagen synthesis, effectively suppressing fibrosis *in vitro*.

#### Promoting degradation of aberrantly deposited ECM components and tissue remodeling

During fibrosis, excessive ECM deposition and cross-linking lead to tissue stiffening and impaired cellular migration. Hydrogels can promote ECM degradation and tissue remodeling through enzyme-responsive or oxidation-responsive designs.

One strategy employs enzyme-loaded hydrogels for localized matrix degradation. For instance, PEG-coated PLGA microspheres encapsulating collagenase or hyaluronidase enable sustained enzyme release, facilitating controlled ECM breakdown and fibrosis reversal [71]. Alternatively, ECM-responsive systems, such as PEG–HA hydrogels containing MMP-cleavable peptides, degrade in high-turnover microenvironments to trigger site-specific release of therapeutic agents, thereby enhancing remodeling specificity and safety compared with exogenous enzyme delivery [70]. In addition, ROS-responsive hydrogels enable stimulus-triggered degradation and drug release under oxidative conditions, reducing collagen accumulation. A representative tyramine-modified gelatin/HA MN system achieved localized SPARC silencing, markedly decreasing collagen deposition with favorable biosafety, offering a promising strategy for scar prevention [107].

#### Modulating the growth and migration of fibroblasts and keratinocytes

The coordinated proliferation and migration of fibroblasts and keratinocytes are essential for skin repair. TEHs can modulate these cellular behaviors to attenuate fibrosis and limit myofibroblast differentiation [121].

Mechanical stiffness critically regulates fibroblast fate, with substrates above ∼20 kPa promoting myofibroblast differentiation, whereas softer matrices (<10 kPa) help maintain a quiescent phenotype [122, 123]. Fibroblast behavior is also influenced by epithelial–mesenchymal communication, as keratinocyte-derived factors such as IL-1 and FGF regulate cell proliferation, migration and matrix remodeling [124, 125]. Because these mechanical and biochemical signals are spatially organized in native skin, stratified hydrogels have been developed to better reproduce this microenvironment. For example, GelMA/HAMA bilayer hydrogels promote directed keratinocyte migration and reduce α-SMA expression, demonstrating how spatial control of matrix properties and biological signals can support tissue stratification and reduce scar formation [126].

#### Modulating the temporal dynamics of immune responses and inflammation

Immune responses play a key role in fibrosis progression, and hydrogels can regulate macrophage polarization and inflammatory signaling. Inflammatory cells and cytokines contribute to skin fibrosis, and hydrogels that modulate macrophage recruitment and activation show therapeutic potential [127].

An effective anti-fibrotic response requires a shift from pro-inflammatory (M1) to anti-inflammatory (M2) macrophages. Immunomodulatory hydrogels can promote this transition by releasing IL-10, IL-4 or IL-13. These factors suppress TNF-α and IL-6 and support tissue repair. For example, a bilayer hydrogel enables sequential release of IL-10 to reduce early inflammation, followed by pro-regenerative factors to support remodeling and macrophage polarization [128].

Hydrogel physical properties also affect immune responses. Softer hydrogels with lower crosslink density promote M2 polarization, while highly crosslinked rigid matrices may enhance inflammatory responses [129]. In addition, polysaccharide-based hydrogels with antioxidant groups (e.g. phenols or selenium-containing components) reduce ROS levels, suppress M1 polarization and improve the inflammatory microenvironment [130, 131].

Fibrosis-related signaling pathways, including the high mobility group box 1/receptor for advanced glycation end-products/nuclear factor kappa B (HMGB1/RAGE/NF-κB) axis [67] and the c-Jun N-terminal kinase (JNK) pathway [116], provide further guidance for hydrogel design. Inhibiting these pathways reduces inflammatory mediator expression and helps limit fibrosis.

### Physical mechanisms

Beyond biochemical regulation, the physical properties of TEHs, including stiffness, elasticity and strain response, play an important role in treating skin fibrosis.

ECM stiffness regulates fibroblast behavior. This mechanical signal is transmitted through the focal adhesion kinase/yes-associated protein/transcriptional coactivator with PDZ-binding motif/myocardin-related transcription factor (FAK/YAP/TAZ/MRTF) pathway to the nucleus, where it controls mechanosensitive gene expression and influences fibroblast phenotype [132]. Mechanical stress contributes to fibrosis by increasing ECM stiffness and activating mechano-transduction and TGF-β1 signaling. This process forms a feedback loop between stiffness and fibrotic signaling. Hydrogels can interrupt this loop by reducing local stiffness or by incorporating inhibitors of lysyl oxidase (LOX), focal adhesion kinase (FAK) or YAP/TAZ, thereby limiting mechanically induced fibrosis [133].

Smart hydrogels with reversible stiffness enable temporal control of the mechanical environment. Photo- and NIR-responsive systems allow tunable mechanical properties [134, 135]. In addition, hydrogels such as thermosensitive poly(amidoamine)-based poly(*n*-isopropyl acrylamide) (PNIPAM-PAA) can regulate tissue tension and ECM stiffness. These materials influence fibroblast phenotype, keratinocyte activity and ECM remodeling, which helps reduce scar formation [136, 137].

Overall, material mechanics act as a controllable factor in fibrosis regulation. Adjusting hydrogel stiffness provides a practical strategy for anti-fibrotic design. A summary of these functional modification strategies, including their key molecular targets and mechanisms of action, is presented in Table 3.

**Table 3:** Key molecular targets and mechanisms of hydrogel-mediated anti-fibrotic therapy.

Mechanism category	Key molecular targets	Hydrogel-mediated effect	Anti-fibrotic outcome
TGF-β/Smad inhibition	TGF-β1, TβR-II/TβR-I; Smad2/3; RBP1, HSP47; AMPK; ROS, Smad7; HDAC	Direct inhibition [13, 108, 118] Indirect regulation [74, 119] Feedback regulation [120] Dual strategy [69]	↓ Myofibroblast differentiation ↓ Myofibroblast apoptosis ↓ Fibrosis progression ↓ Excessive ECM deposition
ECM Degradation	ECM components; MMPs; ROS; SPARC	Direct enzyme delivery [71] Self-regulated MMP-responsive systems [70] ROS-responsive systems [107]	↓ Pathological ECM accumulation ↑ Tissue remodeling ↓ Collagen overproduction ↑ ECM homeostasis
Modulating fibroblasts or keratinocytes	Fibroblasts, keratinocytes; α-SMA; IL-1, FGF; YAP/TAZ	Stiffness-mediated regulation [122, 123] Intercellular signaling modulation [121] Spatial partitioning in stratified hydrogels [126] Keratinocyte-mediated regulation [124, 125]	↓ Fibroblast-to-myofibroblast differentiation ↓ ECM deposition ↓ α-SMA expression ↑ Epidermal regeneration
Immunomodulation	Macrophages (M1/M2 polarization); TNF-α, IL-6, IL-10, IL-4, IL-13; ROS; HMGB1/RAGE/NF-κB axis; JNK pathway	Macrophage polarization regulation [127, 128] Physical property-mediated immunomodulation [129] ROS scavenging [130, 131] Sequential release systems [128] Pathway inhibition [67, 116]	↓ M1 polarization ↑ M2 polarization ↓ Pro-inflammatory cytokines (TNF-α, IL-6) ↑ Anti-inflammatory cytokines (IL-10, IL-4, IL-13) ↓ Inflammatory mediator expression ↑ Regenerative microenvironment
Mechanotransduction	FAK, YAP, TAZ, MRTF; LOX; mechanosensitive transcription factors	Stiffness-mediated signaling modulation; disruption of mechano-feedback loop [132] Dynamic stiffness control via smart hydrogels [134, 135] Multi-cellular regulation [136, 137]	↓ Mechanically driven fibrosis progression ↓ Pathological mechanotransduction signaling ↓ Fibroblast activation ↑ Tissue mechanics restoration

In summary, hydrogels regulate skin fibrosis through coordinated biological and mechanical mechanisms, encompassing TGF-β/Smad inhibition, ECM degradation, immune modulation and disruption of mechanotransduction-driven fibrotic feedback. TGF-β/Smad pathway modulation and macrophage polarization have been validated across multiple *in vivo* models, whereas strategies targeting YAP/TAZ signaling and spatiotemporal multi-cargo delivery remain predominantly supported by *in vitro* evidence, representing a critical gap between mechanistic proof-of-concept and clinical applicability. Future studies should therefore prioritize evaluating these emerging approaches in clinically relevant models to advance the translation of smart hydrogel systems from bench to bedside.

## Hydrogel functional modification for therapeutic enhancement

**Figure 3:**
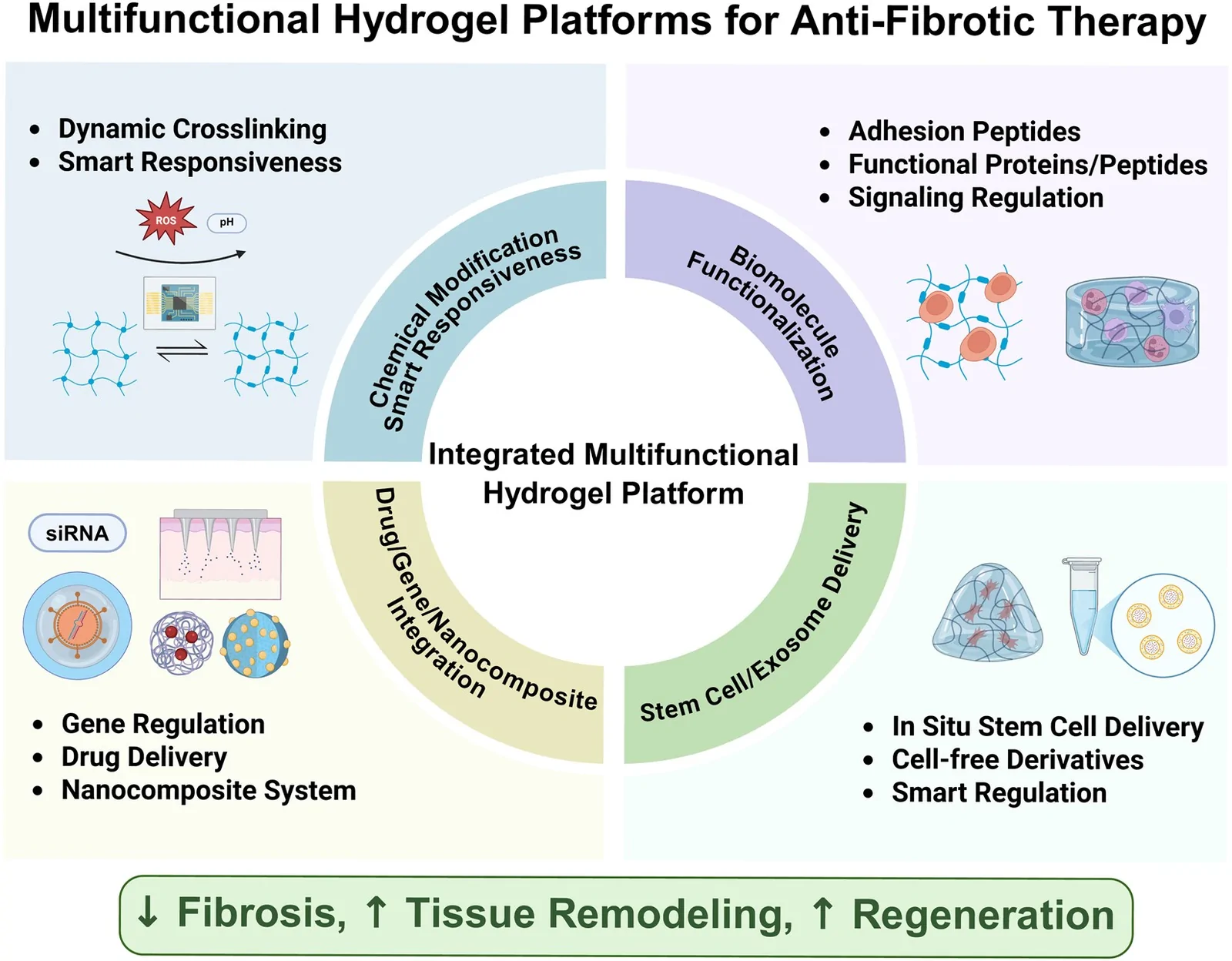
Multifunctional hydrogel platforms for anti-fibrotic therapy. Integrated multifunctional hydrogel platform for anti-fibrotic therapy. Four complementary modules are coordinated: chemical modification with dynamic crosslinking and smart responsiveness; biomolecule functionalization with adhesion peptides and signaling regulators; drug/gene/nanocomposite integration for controlled delivery; and stem cell/exosome delivery for regenerative support. The platform collectively reduces fibrosis, promotes tissue remodeling, and enhances regeneration.

Functional modification enhances the biological activity of TEHs by incorporating bioactive molecules or responsive components that regulate cell behavior and tissue repair. By incorporating chemical, biological or physical design elements, hydrogels can be engineered with specific properties such as targeting capability and stimulus responsiveness. These features expand their potential applications in anti-fibrotic therapy. This section reviews the major functionalization strategies and their underlying mechanisms, including chemical modification, stimulus-responsive design, biomolecule conjugation, drug-nanocomposite systems and stem cell or bioactive factor delivery. As summarized in Figure 3, these strategies can be combined into a multifunctional platform to target the complex pathological mechanisms of skin fibrosis.

### Chemical modification and smart responsiveness

Chemical modification introduces stimulus-responsive elements into TEHs, enabling therapeutic release in response to pathological cues and improving the precision of anti-fibrotic treatment. Common strategies include reversible covalent crosslinking (e.g. Schiff base and Diels–Alder reactions), redox-sensitive bonds, and enzyme-cleavable linkages. These dynamic structures support network reconfiguration and self-healing, helping hydrogels maintain stability under mechanical stress [138]. Such properties support their use as wound dressings or scaffolds after injury for scarless healing [139].

Chemical design can also introduce responsiveness to disease-related signals. ROS-responsive hydrogels contain oxidatively cleavable linkers that degrade in fibrotic microenvironments, enabling localized drug release to reduce oxidative stress and inflammation [140]. Similarly, pH- or enzyme-responsive systems, such as those with MMP-cleavable sequences, degrade in acidic or enzyme-rich regions associated with tissue remodeling, allowing site-specific delivery and reducing systemic effects [141]. Photo-responsive poly(lactic-co-glycolic acid) (PLGA) microcapsule composites provide an alternative strategy by enabling on-demand drug release through light-triggered bond cleavage, thereby improving temporal control over anti-fibrotic therapy [97]. However, the limited penetration of light restricts their application in deep fibrotic tissues, making them more suitable for superficial skin lesions. Future developments may focus on combining photo-responsive systems with NIR activation or thermosensitive degradation mechanisms to improve therapeutic delivery in deeper fibrotic regions [142, 143].

By linking pathological signals, such as ROS levels or MMP activity, to material degradation or drug release, hydrogels can adjust their behavior according to the local microenvironment. Future strategies may integrate multiple responsiveness mechanisms into a single system to improve control [144].

Overall, chemically responsive hydrogels enable drug release to be linked with fibrosis-associated signals, such as elevated ROS levels or MMP activity. As most ROS- and enzyme-responsive systems have been validated mainly *in vitro* or in animal models, future studies should determine whether their activation thresholds match the pathological conditions observed in human fibrotic tissues. Further optimization of responsive chemistry and bioactive payload delivery will be essential for translating these systems into clinically relevant anti-fibrotic therapies.

### Biomolecule functionalization

Incorporating peptides, proteins or polysaccharides into tissue-engineered hydrogels improves cell interaction and signaling properties. These biomolecules are commonly used to enhance cell recruitment, regulate inflammation and limit fibrotic remodeling during wound healing.

For cell recruitment and tissue repair, hydrogels are often functionalized with homing and adhesion motifs. An SDF-1 mimetic peptide (SMP) promotes endogenous stem cell recruitment and epithelialization [145], whereas RGD peptides improve cell adhesion and angiogenesis [72]. In addition, to prevent persistent inflammation from fueling early fibrogenesis, antimicrobial and immunomodulatory sequences are integrated. DP7-modified hydrogels reduce early infection and support stem-cell-mediated repair [146], while C-Histatin-1-functionalized scaffolds promote vascularization and collagen remodeling through sustained peptide release [147].

Other biomolecules directly regulate fibrotic remodeling. PTD-DBM peptides increase type III collagen expression while inhibiting α-SMA-positive myofibroblast differentiation, supporting tissue repair while limiting fibrosis [148]. In addition, peptide functionalization can be combined with other bioactive agents. Co-assembly of peptides with salvianolic acid B (SAB) improves material properties while maintaining antioxidant and anti-fibrotic activity [149]. Similarly, adhesion peptide-modified fiber scaffolds loaded with ginsenoside Rg3 (GS-Rg3) promote early cell migration and later reduce excessive collagen deposition [150].

Overall, biomolecule modification enables controlled regulation of cell behavior, signaling pathways and ECM remodeling. Through the incorporation of bioactive sequences and combinational strategies, hydrogels can better coordinate tissue repair processes and support targeted modulation of fibrotic environments.

### Drug and nanocomposite integration

Drug-nanocarrier integration is a key strategy to enhance the therapeutic efficacy of TEHs. Based on their drug-loading mechanisms, existing systems fall into three main categories: gene-regulatory systems carrying nucleic acid analogues, anti-fibrotic drug delivery systems and nanoparticle-based nanocomposite hydrogels.

#### Gene regulation systems

Nucleic acid analogues, such as siRNAs and viral vectors, can regulate pro-fibrotic pathways, including TGF-β, Wnt and mitogen-activated protein kinase (MAPK). However, their application is limited by rapid degradation, off-target effects and potential toxicity [151–153].

Hydrogel-based delivery systems provide a strategy to address these limitations. Hydrogels can serve as local depots that protect nucleic acids and control their release, improving site retention and therapeutic stability. For example, an alginate hydrogel enables sustained adenovirus delivery to inhibit Wnt signaling [154]. siRNA targeting TGFβ1-337 reduces collagen deposition in scars [155]. A PUMA siRNA hydrogel suppresses radiation-induced ROS/TGF-β signaling and helps restore skin homeostasis [156]. Protein-based regulatory strategies have also shown promising therapeutic potential. For example, a thermosensitive F127 hydrogel loaded with a truncated latency-associated peptide (SE-tLAP) and enhanced by the SPACE transdermal delivery peptide was reported to suppress TGF-β/Smad signaling and reduce bleomycin-induced skin fibrosis *in vivo* [77]. However, most hydrogel-mediated regulatory approaches, including both nucleic acid- and protein-based interventions, remain at the preclinical stage and have primarily been validated in cell and animal models. Further studies are therefore needed to evaluate their long-term biosafety, reproducibility, and manufacturing consistency before clinical translation.

#### Anti-fibrotic drug delivery systems

Hydrogels provide localized platforms for anti-fibrotic drug delivery and improve targeting compared with systemic administration. By retaining drugs at fibrotic sites, they increase local bioavailability, prolong drug action and reduce off-target effects. Based on therapeutic strategy, these systems can be grouped into three categories: delivery of signaling pathway inhibitors, local delivery of systemic anti-fibrotic drugs and multi-target composite systems.

Hydrogels can locally release signaling inhibitors to reduce scar formation. For example, systems targeting the Hippo pathway (YAP/TAZ) include an arsenic trioxide-loaded hydrogel (ATO@CS/SA) that promotes scarless healing [157] and a kaempferol-loaded F-127 thermo-responsive hydrogel that suppresses fibrosis through Hippo pathway regulation [76]. Photo-responsive hydrogels loaded with photosensitizers (VP) regulate TGF-β/Smad2 signaling after light activation and support scar-free repair [116, 158, 159].

Hydrogels also enable local delivery of drugs originally developed for systemic fibrosis. For example, pirfenidone (PFD) delivered by an SA/BG-SACM-PLGA hydrogel reduces TGF-β1 expression and scar formation [102]. Sitagliptin (SITA) inhibits TGF-β1 and the DPP-4 pathway to alleviate fibrosis [30]. SBA and astragaloside IV, delivered through SAB-TAT-LIP and SLN-gel systems, also show anti-fibrotic effects [160–162]. These studies indicate that hydrogel delivery improves drug stability and local therapeutic activity.

Multi-target composite systems further expand hydrogel-based therapy. For instance, stratifin (SFN) facilitates keratinocyte‑fibroblast communication to guide ECM remodeling [163]. The epigenetic modulator trichostatin A (TSA) inhibits HDAC to reduce collagen production [69]. Meanwhile, GS-Rg3 functions to balance angiogenesis and collagen turnover, promoting regeneration without scarring [150]. Together, these multi‑target composites exert comprehensive anti‑fibrotic activity through coordinated regulation of inflammation, signaling and matrix metabolism.

#### Nanocomposite systems

To address the oxidative stress and abnormal ECM remodeling involved in skin fibrosis, nanocomposite hydrogels incorporate functional nanoparticles to achieve local antioxidant effects and regulate matrix reconstruction. Organic-inorganic hybrid systems can reduce oxidative damage through direct radical scavenging; for example, lipid-based, plant-derived or metal oxide components, including omega-3, chamomile extract and Ag-ZnO/AgO clusters, reduce inflammatory cell infiltration and promote MMP-2/9-mediated matrix degradation [33, 164, 165]. In addition, polymer-based nanocarriers provide controlled delivery of bioactive molecules to regulate scar formation. For example, polydopamine-encapsulated asiaticoside reduces excessive fibrotic deposition [88], obestatin nanoreactors promote collagen and VEGF expression [32] and PLGA-based insulin microparticles regulate pro-inflammatory macrophage responses and guide collagen remodeling toward a more regenerative fiber organization rather than a dense fibrotic pattern [166]. Compared with enzyme-based systems, these nanoparticle platforms provide more stable and sustained anti-fibrotic effects in the protease-rich wound environment.

### Stem cell and bioactive factor delivery

Stem cell therapy represents a major advancement in anti-fibrotic medicine. However, traditional cell transplantation is heavily restricted by poor survival and uncontrolled differentiation in hostile fibrotic niches. Merging TEHs with stem cell therapies yields a synergistic platform where the polymer network functions as an active, instructive niche. In contrast to the conventional function of physical carriers, smart hydrogel systems have been shown to interact dynamically with transplanted cells or their secretomes. These platforms buffer early inflammatory stress, enhance long-term cell retention and deliver precise physicochemical cues that direct anti-fibrotic cell behaviors.

#### Enhancing *in situ* retention and viability of living stem cells

To maximize the efficacy of living stem cells, hydrogels must establish a protective and biomimetic microenvironment at the fibrosis site. By replicating natural ECM geometries, these platforms support post-transplantation cell viability and attenuate subsequent scar formation. For instance, small intestinal submucosa (SIS) ECM hydrogel patches substantially prolong the localized survival of adipose-derived stem cells (ADSCs) [167], while specific polyhydroxyalkanoate (PHBV) scaffolds maintain a moist environment that limits wound contraction and works synergistically with ADSCs to promote ordered angiogenesis while downregulating TGF-β1 expression [168]. Furthermore, encapsulating mesenchymal stem cells (MSCs) within thermosensitive alginate microspheres physically insulates cells from mechanical stress at dynamic wound beds, prolonging the release of therapeutic factors like IL-10 [169]. To enhance cell-matrix engagement, incorporating functional motifs such as RGD peptides within PEG-HA networks or fabricating interconnected macropores in GPVA5_CNP hydrogels significantly improves cell adhesion, proliferation and anti-fibrotic phenotype retention [95, 170]. Additionally, composite systems like porous hydrogels carrying the antimicrobial peptide DP7 together with placental MSCs (PMSCs) can simultaneously suppress infection, modulate acute inflammation, and enhance tissue remodeling to achieve scar-free healing [146].

#### Smart-responsive cues modulating stem cell anti-fibrotic behaviors

Beyond providing basic structural housing, smart hydrogel systems leverage environmental responsivity and temporal programming to actively direct stem cell phenotypes *in vivo*. Because skin fibrosis evolves dynamically over time, hydrogels must adapt their physicochemical properties to match the shifting demands of the healing niche. For instance, thermo-responsive sECM-MC matrices undergo rapid phase transitions at body temperature, successfully immobilizing transplanted cells within deep dermal margins while steadily releasing bioactive factors to promote early epithelialization and controlled vascularization [171]. Furthermore, advanced materials can establish sequential delivery programs to match the biological cascades of tissue repair. For example, a dual-layer BSSPD hydrogel has been demonstrated to achieve chronologically programmed cargo release by dispatching pro-angiogenic sEVs during the acute phase and delaying anti-hyperplastic sEVs to the remodeling phase. This temporal coordination effectively guides stem cells through a more organized and scarless healing trajectory [172].

#### Cell-free engineered systems for stem cell derivatives

To avoid the risks and transplantation limitations of living cells, smart hydrogels have been developed as cell-free delivery platforms for stem cell derivatives, including conditioned media (CM), paracrine proteins and exosomes. Polysaccharide-based hydrogels encapsulated with adipose-derived stem cell concentrated conditioned medium (ADSC-CM) exhibit a dose-dependent reduction in the myofibroblast marker α-SMA, improving downstream collagen remodeling [173]. For severe full-thickness burns where deep appendages are lost, the use of PEG-based hydrogels engineered for the sustained release of MSC paracrine proteins (MSC-PP) from three-dimensional dynamic cultures significantly stimulates functional skin appendage regeneration [174]. Crucially, delivering human umbilical cord MSC-derived exosomes (hUCMSC-exosomes) via smart hydrogels represents a promising cell-free therapeutic strategy. These systems facilitate full-thickness cutaneous restoration by ensuring prolonged cargo bioactivity, accelerating re-epithelialization and driving precisely controlled tissue remodeling to minimize fibrotic progression [79, 80, 175, 176].

Collectively, the evidence reviewed in this section remains predominantly preclinical. Future studies should systematically compare the translational feasibility of living stem cell transplantation versus cell-free derivatives, such as exosomes and conditioned media. This comparison should consider factors including immunogenicity, regulatory classification and manufacturing complexity, to guide the prioritization of these strategies toward clinical evaluation.

## Conclusion and perspectives

Skin fibrosis is a pathological process arising from abnormal wound healing, chronic inflammation or radiation injury. It is characterized by excessive ECM deposition and disrupted tissue architecture. Current clinical treatments, including corticosteroid injections, surgical excision, pressure therapy and radiation therapy, face substantial limitations. These include high recurrence rates, significant side effects and an inability to modulate the pathological microenvironment effectively.

Most existing TEHs still rely on fixed structures and passive drug delivery. Their degradation profiles and release kinetics are determined at the time of implantation and cannot adjust to dynamic changes in the fibrotic microenvironment, such as shifts in pH, enzyme activity or mechanical stress. Furthermore, most systems depend on a single therapeutic mechanism rather than integrating mechanical modulation, immunomodulation and regenerative support. This restricts their effectiveness in addressing the multifactorial pathophysiology of skin fibrosis.

Rather than pointing to a single optimal design, the evidence reviewed here suggests that hydrogel design should be matched to the clinical context of fibrosis. In acute or post-surgical settings, where intervention windows are short and inflammatory activity is high, hydrogels that respond rapidly to stimuli and degrade readily are likely to be the most practical option. For chronic fibrotic conditions, where pathological networks are already established, multifunctional systems integrating drug-, gene- or cell-based therapies may be required to achieve sustained therapeutic effects. For deep or irregularly shaped lesions, injectable and minimally invasive delivery formats are likely to take priority, ensuring procedural feasibility while accommodating the functional complexity required for effective treatment. This context-dependent approach to design selection, rather than a uniform optimization strategy, is likely to better reflect the heterogeneous clinical needs of skin fibrosis.

Despite these design principles, the clinical translation of smart hydrogels for skin fibrosis remains limited by several challenges. Most efficacy data originate from acute or subacute rodent wound models, which do not adequately reflect the chronic and heterogeneous nature of human fibrotic diseases. Beyond this limitation, several key issues warrant greater attention. First, the activation thresholds of responsive hydrogels should be better matched to the actual levels of ROS, enzymes and other pathological cues found in human fibrotic tissues. Second, future systems should move beyond static stiffness matching and achieve mechanical adaptation throughout different stages of tissue repair. Third, more direct comparisons between stem cell-based and cell-free approaches are needed to balance therapeutic efficacy with manufacturing and regulatory feasibility. Finally, advanced architectures such as multilayer patches and microneedles will require scalable production methods, robust sterilization strategies and reliable quality control before clinical implementation becomes feasible.

In summary, intelligent hydrogels have shown considerable potential for the treatment of skin fibrosis. Future progress will depend not only on improving therapeutic efficacy but also on addressing key translational challenges, including pathological cue matching, dynamic mechanical regulation, bioactive cargo selection and scalable manufacturing. Tackling these issues will be essential for advancing anti-fibrotic hydrogels from experimental systems toward clinical applications.
